# COMPASS: Computations for Orientation and Motion Perception in Altered Sensorimotor States

**DOI:** 10.3389/fncir.2021.757817

**Published:** 2021-10-15

**Authors:** Victoria G. Kravets, Jordan B. Dixon, Nisar R. Ahmed, Torin K. Clark

**Affiliations:** ^1^Bioastronautics Laboratory, Ann and H.J. Smead Department of Aerospace Engineering Sciences, University of Colorado Boulder, Boulder, CO, United States; ^2^COHRINT Laboratory, Ann and H.J. Smead Department of Aerospace Engineering Sciences, University of Colorado Boulder, Boulder, CO, United States

**Keywords:** sensory conflict, Bayes rule, astronaut, gravity, adaptation, cognitive modeling

## Abstract

Reliable perception of self-motion and orientation requires the central nervous system (CNS) to adapt to changing environments, stimuli, and sensory organ function. The proposed computations required of neural systems for this adaptation process remain conceptual, limiting our understanding and ability to quantitatively predict adaptation and mitigate any resulting impairment prior to completing adaptation. Here, we have implemented a computational model of the internal calculations involved in the orientation perception system’s adaptation to changes in the magnitude of gravity. In summary, we propose that the CNS considers parallel, alternative hypotheses of the parameter of interest (in this case, the CNS’s internal estimate of the magnitude of gravity) and uses the associated sensory conflict signals (i.e., difference between sensory measurements and the expectation of them) to sequentially update the posterior probability of each hypothesis using Bayes rule. Over time, an updated central estimate of the internal magnitude of gravity emerges from the posterior probability distribution, which is then used to process sensory information and produce perceptions of self-motion and orientation. We have implemented these hypotheses in a computational model and performed various simulations to demonstrate quantitative model predictions of adaptation of the orientation perception system to changes in the magnitude of gravity, similar to those experienced by astronauts during space exploration missions. These model predictions serve as quantitative hypotheses to inspire future experimental assessments.

## Introduction

In everyday life, we must reliably perceive self-motion and orientation while being capable of adapting to novel stimuli, changing environments, or changes to peripheral sensory organs (e.g., from childhood development, aging, or injury). Here, we explore the computations that may be necessary to produce estimates of worldly parameters, when those parameters change (or even may be unknown to begin with). We specifically focus on how humans adapt their orientation perception systems during gravity transitions, such as those experienced by astronauts (Paloski et al., 2008). While our initial modeling implementation and simulations are specific to gravity transitions, the computational mechanisms proposed may be broadly applied to sensorimotor adaptation to various changing environments or peripheral sensory organ states.

Upon entering microgravity, astronauts visiting Low Earth Orbit initially experience space motion sickness (Lackner and Dizio, 2006) and disorientation (Paloski et al., 2008). With extended exposure, the brain reinterprets sensory information, adapting to the new environment, and impairment is reduced (Shelhamer, 2015). While all astronauts eventually adapt to the microgravity environment (Shelhamer, 2015) these adaptations produce sensorimotor impairment upon return to Earth. This includes postural (Wood et al., 2015) and locomotion deficits (Mulavara et al., 2018), misperceptions of spatial orientation (Clement and Wood, 2014), altered eye movements (Clement, 1998), manual control decrements (Merfeld, 1996), motion sickness (Lackner and Dizio, 2006; Reschke et al., 2018), and ataxia (Paloski et al., 1993). When returning to Earth, a ground support crew mitigates astronaut sensorimotor impairment operationally helping enable mission success/safety. However, sensorimotor impairment may have catastrophic impacts on future moon or Mars landings where astronauts may have a more active piloting role and will not have a ground support crew to assist after landing, at least initially (Clark, 2019).

The potential mechanisms of sensorimotor adaptation to altered gravity remain conceptual (Young et al., 1984; Parker et al., 1985; Merfeld, 2003; Clark, 2019), limiting our understanding and ability to predict and mitigate impairment. Specifically, it has often been suggested that changes in the physical environment, such as an astronaut exposed to a gravity transition, require a reinterpretation of sensory information (Young et al., 1984). Conceptually, it has been proposed that this occurs by considering “sensory conflict” (i.e., the difference between actual and expected sensory measurements) (Oman, 1998). If sensory conflict is large and sustained, the central nervous system (CNS) will reinterpret sensory information to reduce sensory conflict, thereby effectively adapt to the new environment (Oman, 1982). While this hypothesis is accepted to some degree, it remains conceptual. Specifically, the detailed computations required by neural systems have not been specified, preventing numerical implementation, and thus the theory cannot be rigorously tested. In fact, the lack of quantitative specificity often makes these types of conceptual hypotheses excessively flexible in their predictions.

Here, we have implemented a computational model for how the orientation perception system adapts to transitions in the magnitude of gravity. In summary, it hypothesizes that the CNS uses: (1) internal models of worldly and sensory physics to produce expected afferent measurements (i.e., transduction of actual orientation) and resultant sensory conflict signals; (2) parallel, alternative hypotheses for the internal magnitude of gravity, each of which produce a set of different sensory conflict signals; (3) Bayes rule for sequentially computing the posterior probability of each hypothesis based upon the likelihood of the overall sensory conflict observed; (4) a computed probability of each alternative hypothesis to yield an estimated internal magnitude of gravity, over time, which is then used to process sensory information and produce perceptions of passive self-motion and orientation. Within this paper, we do not consider the specific brain regions or the neurotransmitter/neural network implementations involved biologically, but instead focus on the potential cognitive computations that may be needed to adapt to altered sensorimotor states (environmental, peripheral organ state). Once implemented, we simulated the computational model to make quantitative predictions for how humans may adapt to various gravity transitions. While the exact neural mechanisms for sensorimotor adaptation to altered gravity remain difficult to identify experimentally, by implementing a computational model we can explore the types of computations necessary to enable such adaptation, as well as produce novel quantitative hypotheses to motivate future experimental investigation.

## Background and Existing Work

**FIGURE 1:**
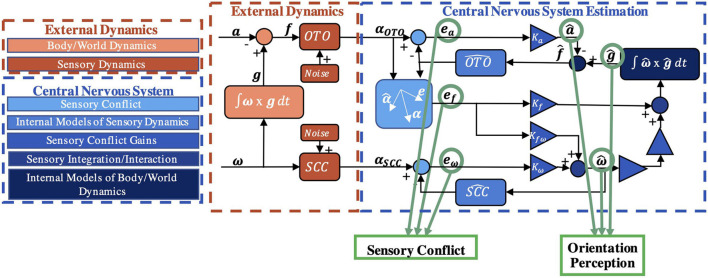
Vestibular portion of the observer model. The model input is a time history of physical self-motion (linear acceleration **a**, angular velocity ω, gravity **g**) and predicts dynamic spatial orientation perception ($\hat{\text{a}}$, $\hat{\omega }$, $\hat{\text{g}}$, where the “hat” indicates a perception) driven by sensory conflict cues (**e_a_**, **e_f_**, **e_ω_**) resulting from comparisons of incoming afferent signals (α_**OTO**_, α_**SCC**_) to the expectation (Merfeld et al., 1993a; Clark et al., 2019). Bold denotes a 3D vector.

It has previously been hypothesized that to interpret sensory information and properly perceive spatial orientation here on Earth (Merfeld et al., 1999), the CNS uses *internal models* [neural systems that replicate the behavior/dynamics of physical systems (Von Holst and Mittelstaedt, 1950; Reason, 1978; Mittelstaedt, 1983; Merfeld et al., 1999; Tin and Poon, 2005)]. This hypothesis has been formalized into computational models, such as the “observer” model, for spatial orientation (Merfeld et al., 1993b; Merfeld and Zupan, 2002; Zupan et al., 2002; Clark et al., 2019). In the observer framework (Luenburger, 1971), actual orientation, produced from body/world dynamics, is transduced by noisy sensors with dynamics [e.g., semicircular canals have high-pass filter dynamic characteristics in which low frequency rotations are not transduced well (Goldberg and Fernandez, 1971)] to yield sensory afference (measurements). Internal models of body/world dynamics and the sensory dynamics produce an expected afference (Merfeld et al., 1993a). This is compared to the actual sensory afference to yield “sensory conflict” (Oman, 1982). In estimation theory, this difference between actual and expected measurements is known as the “innovation” (Kalman, 1960; Kalman and Bucy, 1961). Neural populations in the vestibular nuclei (Roy and Cullen, 2004; Jamali et al., 2009) and in the cerebellum (Brooks and Cullen, 2009, 2013) behave similar to that expected of this sensory conflict signal, supporting this model formulation (Oman and Cullen, 2014). Within observer, sensory conflicts are weighted to drive central estimates of orientation perception (Merfeld et al., 1993a; Clark et al., 2019). In a Kalman filter (Kalman, 1960; Kalman and Bucy, 1961), the innovation is multiplied by the Kalman gain to update state estimates (Karmali and Merfeld, 2012; Selva and Oman, 2012). As shown in Figure 1, the observer model has been formalized for vestibular processing (Merfeld et al., 1993b), as well as visual-vestibular interaction (Newman, 2009; Clark et al., 2019) (not further considered here). Inputs of angular velocity (ω) and linear acceleration (**a**) stimulate the otoliths (OTO) and semicircular canals (SCC) producing sensory measurements (**α_**OTO**_** and **α_**SCC**_**), which are compared to expectations to yield sensory conflict signals (**e_**a**_**, **e_**f**_**, **e_ω_**). The weights (K_*a*_, K_*f*ω_, K_ω*f*_, K_ω_) are free parameters which have been previously defined, yielding perceptions of linear acceleration ($\hat{\text{a}}$), gravity ($\hat{\text{g}}$) (i.e., tilt), and angular velocity ($\hat{\omega }$). Notably, the model has been well-validated by using a range of motion paradigms in 1 Earth *g* (Clark et al., 2019) to predict self-orientation and motion perceptions measured experimentally.

However, sensory information [e.g., from the otoliths (Fernandez and Goldberg, 1976)] is altered following a gravity transition (Clark et al., 2015b; Galvan-Garza et al., 2018), making the internal models inappropriate (Clark et al., 2015c). This results in misperceptions of orientation (Clement et al., 2007; Clement and Wood, 2012; de Winkel et al., 2012) and large, sustained sensory conflict. Sensory conflict is thought to drive a reinterpretation of internal models (Brooks et al., 2015; Carriot et al., 2015), reducing misperceptions. Yet, to date, this conceptual hypothesis has not been formalized into a computational model. Notably, the computations performed by the CNS necessary to update the internal models using the sensory conflict signals remain undefined.

Here, we leverage the well-validated observer model for vestibular processing and spatial orientation perception (Merfeld et al., 1993a; Clark et al., 2019) as the basis of our computational model for adaptation to gravity transitions. While the observer model has been used to predict orientation perception in altered gravity environments (Clark et al., 2011, 2015c; Vincent et al., 2016; Clark and Young, 2017), here we extend this base model to consider *adaptation* to gravity transitions. Specifically, multiple observer models are processed in parallel, each with a different alternative hypothesis for the internally estimated magnitude of gravity. With the same incoming sensory information (e.g., from the otoliths) this produces different alternative sensory conflict signals. Conceptually, the smallest overall sensory conflict will tend to be produced by the alternative hypothesis for the internally estimated magnitude of gravity which is closest to the actual magnitude of gravity following a transition. These computations enable the CNS to identify changes in the actual magnitude of gravity and update internal models accordingly based only upon processing sensory information.

## Summary of Proposed Theory

While the implementation example presented in the subsequent sections applies to CNS estimation of the magnitude of gravity, the basic theory we propose can be applied generally to any aspect of sensorimotor adaptation using a human orientation perception model that utilizes the concept of sensory conflict. While we use the observer model in our preliminary implementation, the procedure only necessitates a metric of sensory conflict to drive the adaptation process. We posit that the CNS strives to minimize the amount of sensory conflict experienced by sequentially updating its estimate of the parameter of interest.

To determine what value of a given parameter will minimize sensory conflict, we assert the CNS evaluates alternative hypotheses for the value of that parameter. Determining the sensory conflict that would be achieved with different parameter values requires multiple, parallel versions of the sensory processing, each with their own value of the parameter. Each of these parallel alternatives is simulated with the incoming sensory measurements, but due to the different parameter values, each will produce different expected sensory measurements and thus different sensory conflict signals. Conceptually, the parallel, alternative hypothesis with the parameter estimate that best matches the current, actual parameter level will tend to yield the lowest sensory conflict, limited by sensory noise.

For parameters that relate to multiple sensory conflict signals, such as how accelerations and rotations are required for the CNS to determine an accurate perception of gravity, we propose that the CNS weights and normalizes disparate conflicts by the typical reliability of the sensory signals (e.g., biological noise, observability of perceptual state) in order to produce a unidimensional metric of conflict for a given alternative hypothesis. With this statistic, we hypothesize that the CNS uses mechanisms akin to Bayes rule to compute the posterior probability of each hypothesis based on the likelihood of the overall conflict observed, given new sensory measurements over time.

A central estimate of the parameter of interest is computed based on the probability distribution of the hypotheses’ posteriors. We suggest the CNS uses this “best estimate” to process sensory information through a central internal model responsible for producing perceptions of self-motion and orientation that are used for sensorimotor action. Taken all together, we theorize the necessary types of cognitive-based Computations for Orientation and Motion Perception in Altered Sensorimotor States (COMPASS) related to physical environmental factors and peripheral sensory changes.

## Computational Implementation

**FIGURE 2:**
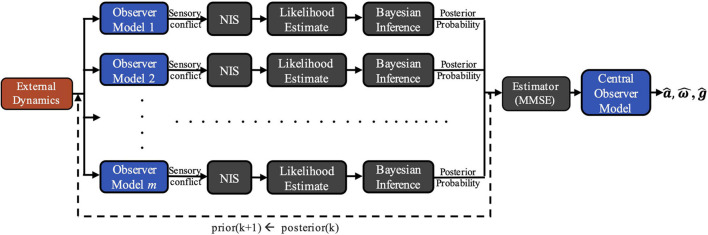
COMPASS model framework. External dynamics are inputted into m parallel observer models, each incorporating an alternative hypothesis for the magnitude of gravity, |$\hat{\text{g}}$|. Differences between expected and actual sensory afferent signals in each observer generate sensory conflict, allowing for Bayesian posterior probability updates for each gravity hypothesis after being weighted and normalized by the Normalized Innovation Squared (NIS) function. A central estimate is derived from the posterior probability distribution and is inputted into a final Central Observer Model, yielding perceptual estimates of $\hat{\text{g}},\hat{\text{a}},\mathrm{and}\hat{\omega }$.


To demonstrate the viability of this theory, we developed a computational model of vestibular adaptation to gravitational transitions. In the preliminary implementation of the overall model, *m* observer models are run in parallel, each incorporating an alternate hypothesis, *H*_*j*_, of the internal estimate of the magnitude of gravity, |$\hat{\text{g}}$|, that leads to distinct sensory conflict (Figure 2). As seen in Figure 1, there are three sensory conflict signals (**e**_**a**_, **e**_**f**_, **e_*ω*_**), each of which is three-dimensional and vary in units. To combine these disparate, multi-dimensional signals into a single, useful metric of sensory conflict for each hypothesis, we use a statistic from adaptive estimation theory called Normalized Innovation Squared (NIS) (Bar-Shalom et al., 2001; Chen et al., 2018):

$$\epsilon_{k}^{j}={\left({\text{e}}_{k}^{j}\right)}^{T}{\left(S\right)}^{-1}{\text{e}}_{k}^{j}$$

where S=[σa2000σf2000σω2], ${\text{e}}_{k}^{j}=\left[\begin{array}{c} ||{\text{e}}_{k,a}^{j}||\\ ||{\text{e}}_{k,f}^{j}||\\ ||{\text{e}}_{k,\omega }^{j}|| \end{array}\right],$ *j* is the alternative hypothesis, and *k* is the time step.

The NIS stochastic objective function characterizes the performance of estimation filters when only sensor data (e.g., otolith and SCC afferents) are available, which represents the CNS learning worldly parameters without (subconscious) access to the ground truth. This is achieved by weighting each sensory conflict signal by its reliability, which in this case, is related to the inverse of the estimated biological noise covariance (σa2, σf2,σω2). In this way, larger sensory conflict errors in signals that are generally well perceived (e.g., low noise, observable) contribute to higher overall values of the NIS statistic, indicative of worse estimation performance. This is reasonable when the primary source of sensory conflict is measurement noise. However, in our simulations sensory conflict is also very much due to the passive motion being experienced (even prior to any gravity transition). Thus, we incorporate a scaling factor K_*s*_ to the **S** matrix (see Supplementary Table 1). This can be thought of as related to the magnitude of “process noise” from passive motions, as well as multi-state contributions to a measurement. For simplicity, the errors in this implementation are treated as uncorrelated, such that the **S** matrix is diagonal; however, in principle correlations in sensory conflicts could be accounted for with off-diagonal elements being non-zero. While it modulates the adaptation rate, K_*s*_ is held constant in all of our simulations and is a free parameter to be defined with future experimental evidence that quantify empirical rates of adaptation. Therefore, the example simulations presented here do not specify values of Time on the x-axes. The scaling on time depends upon several meaningful factors that we explore below, but also on computational assumptions like the granularity of alternative hypotheses, the time step for numerical integration, and whether the orientation perception model vs. the update to the internal magnitude of gravity happen with the same synchronous time steps.

Once the unidimensional, unitless NIS statistic is computed for a given hypothesis, *H*_*j*_, a likelihood for the magnitude of gravity can be produced. Mathematically, under linear-Gaussian assumptions, it is given by the measurement likelihood:


$$p\left(y_{k}|H_{j}\right)=N\left[{\text{e}}_{k}^{j};0;\text{S}\right]=\frac{1}{\sqrt{{\left(2\pi \right)}^{n}\left|S\right|}}e^{-\frac{1}{2}\epsilon_{k}^{j}}$$

where N[${\text{e}}_{k}^{j};$0; **S**] is a Gaussian probability distribution function with a mean of 0 and variance **S**, evaluated at ${\text{e}}_{k}^{j}$ (the measurement error for alternative hypothesis *j* at time point *k*), and *n* is the length of ${\text{e}}_{k}^{j}$, in this case involving three sensory conflict signals. Conceptually, the measurement error should be normally distributed with variance **S**. Thus, the probability that a specific error is observed is the probability at that location on the normal distribution, which is a function of the corresponding NIS for the error.

Likelihood calculations are performed for each of the *m* discrete, alternate hypotheses. The posterior probability of each gravity hypothesis is then computed using Bayes rule:


$$p\left(H_{j}|y_{k}\right)=\frac{p\left(y_{k}|H_{j}\right)p\left(H_{j}\right)}{p\left(y_{k}\right)}$$

Where the prior for each hypothesis, *p*(*H*_*j*_), at time step *k* is the posterior from the previous time step, *p*(*H*_*j*_ | *y*_*k*−1_). Again, in our model, each *H*_*j*_ is an alternative internal magnitude of gravity (e.g., *H*_1_: |$\hat{\text{g}}$| = 1.0 *g*) and *y* is the sensory measurement (e.g., from the otoliths and semicircular canals). *p(y_*k*_)* is the marginal likelihood that the measurement *y* was observed. With a finite number (*m*) of alternative hypotheses, the marginal likelihood can be computed as:


$$p\left(y_{k}\right)=p\left(y_{k}|H_{1}\right)p\left(H_{1}\right)+p\left(y_{k}|H_{2}\right)p\left(H_{2}\right)+\ldots +p\left(y_{k}|H_{m}\right)p\left(H_{m}\right)$$

Finally, using the probability distribution of the alternative hypotheses, a central estimate of |$\hat{\text{g}}$| is calculated. For preliminary implementation, a Minimum Mean Squared Error (MMSE) estimator is used as the central estimator and is calculated as follows:


$$H_{MMSE}=\sum_{j=1}^{m}H_{j}*p\left(H_{j}|y_{k}\right)$$

The MMSE estimate acts as the |$\hat{\text{g}}$| value in a final (*m*+1), Central Observer model, while the *m* original observer models maintain their hypothesized |$\hat{\text{g}}$| values in each time step. The Central Observer Model represents the CNS’s currently “accepted” internal model, and generates the estimates of percepts $\hat{\text{g}}$, $\hat{\text{a}}$, and $\hat{\omega }$ at each time step. Within this, we have included a recent published enhancement to the observer model (Clark et al., 2015c) which incorporates differential weighting of the otolith stimulation in the utricular plane vs. perpendicular to it (i.e., primarily stimulating the saccule). This enhancement allows for the model to properly predict overestimation of roll tilt in hyper-gravity (and underestimation in hypo-gravity), which is pertinent to the gravity transitions being simulated.

## Example Simulations of the Implemented Computational Model

**FIGURE 3:**
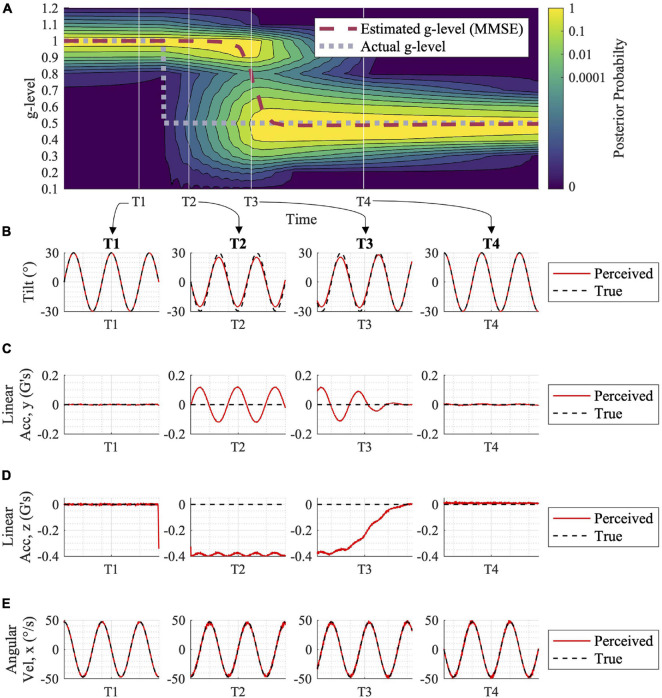
Adaptation to gravity transition with associated expected tilt, acceleration, and angular velocity perceptions. **(A)** The motion profile utilized in this simulation was a sinusoidal, head-centered roll-tilt at 30° amplitude and 0.25 Hz frequency. Following a shift in gravity from 1 to 0.5 *g* (gray dashed line in **A**), the posterior probability distribution shifts, and the central estimator adjusts to the new gravity level over time (red dashed line). This adaptation process is driven by sensory conflict signals for each alternative hypothesis for the internal magnitude of gravity (not shown here, but see Supplementary Figure 1). At time points T1, T2, T3, and T4, **(B–E)** show the Central Observer Model-predicted perceived (red) vs. true (black) **(B)** tilt, **(C)** linear acceleration (y component), **(D)** linear acceleration (z component), and **(E)** angular velocity (x component, as the others are essentially zero). While at T1, prior to the gravity transition, motion is accurately perceived. Misperception occurs at times T2 and T3 (albeit, less misperception) while the model is undergoing the adaptation process. By T4, when the adaptation is complete, motion is accurately perceived in 0.5 *g*.

**FIGURE 4:**
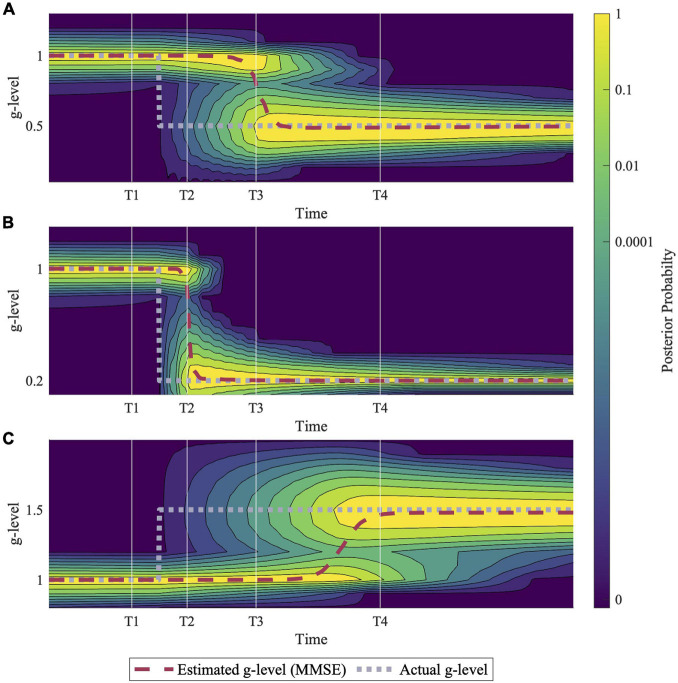
Example simulations of hypo- and hyper- gravity adaptation scenarios. **(A)** Shows an adaptation to 0.5 *g*, **(B)** shows adaptation to 0.2 *g*, and **(C)** shows adaptation to 1.5 *g*. Simulation results suggest that adaptation to hyper-gravity (1.5 *g*) from 1 *g* may take longer than adaptation to hypo-gravity (0.5 *g*, 0.2 *g*). Each simulation was completed using a sinusoidal roll-tilt (30°, 0.25 Hz) motion profile.

**FIGURE 5:**
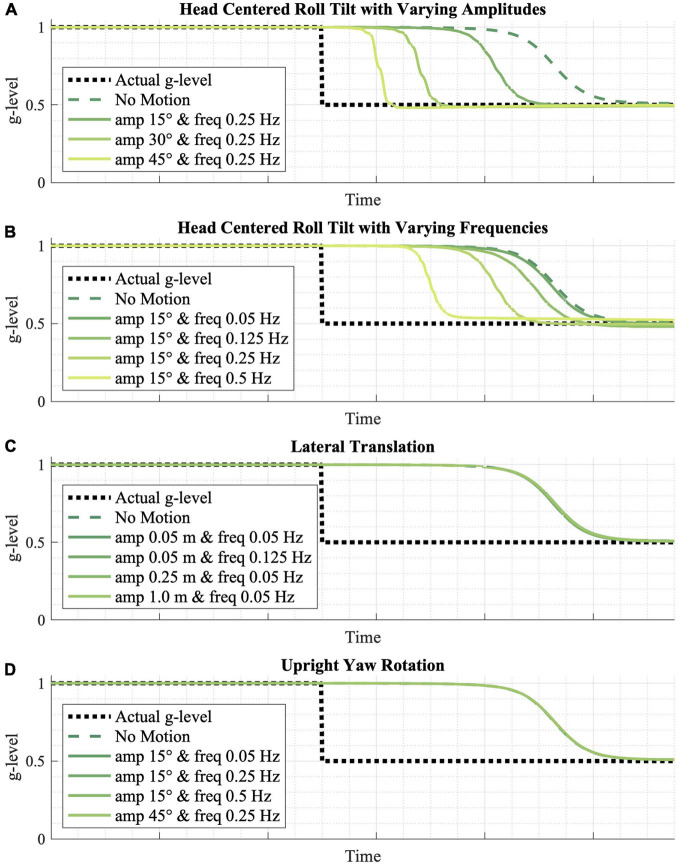
The effect of various passive motion profiles on gravity adaptation. **(A,B)** Passive roll-tilt, **(C)** translation, and **(D)** upright yaw rotation motion profiles were simulated, predicting that varying frequencies and magnitudes of upright yaw and lateral translation yield no effect on the gravity adaptation process, while higher frequency and higher amplitude roll-tilt motion profiles speed up the adaptation process.

**FIGURE 6:**
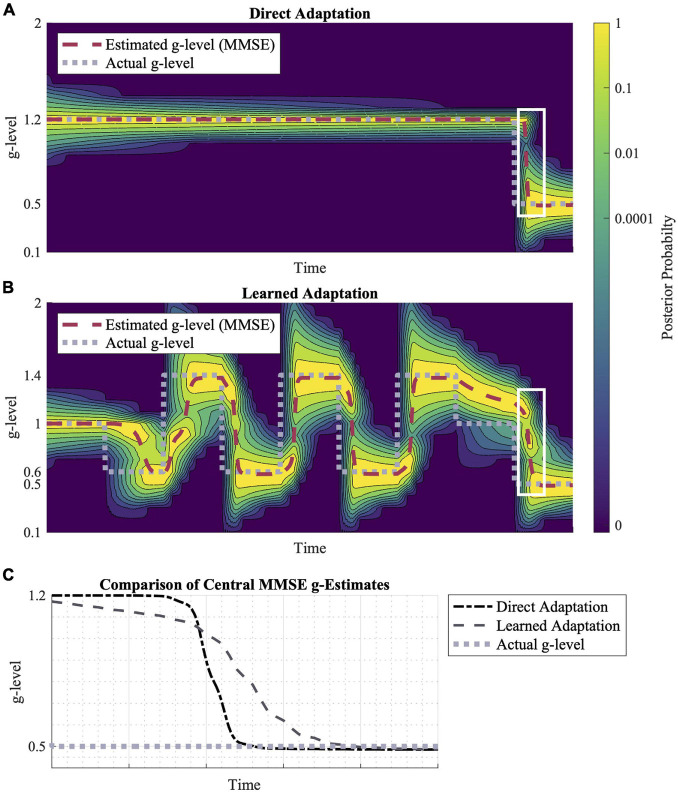
Direct adaptation vs. learned adaptation scenarios. **(A)** Direction adaptation from 1.2 to 0.5 *g*. **(B)** Learned adaptation using oscillating “training” gravitational levels to create a wider probability distribution leading to a final gravity level of 0.5 *g*. **(C)** A closer look at the white highlighted regions in **(A,B)**, shows their relative adaptation rates to 0.5 *g*. While the learned adaptation scenario begins adapting first, the direct adaptation converges to 0.5 *g* faster. Inputted motion profiles were sinusoidal, head-centered roll-tilt (amplitude, 30°; frequency, 0.25 Hz).

Using the computational implementation methods described above, we simulated vestibular adaptation to discrete gravitational transitions during various passive motion profiles. Each simulation results in a posterior probability distribution for the alternative hypotheses, an MMSE estimate, and associated predicted perceptions (Figure 3) over time. Again, note that the units of time are arbitrary until experimental evidence better defines time constants, and thus are not shown in the results. While holding all model parameters constant, the adaptation rate is dependent on the actual gravity profiles (Figure 4), motion profiles (Figure 5), and the distribution of prior probabilities produced by previous gravity transitions at the time point of a subsequent gravity transition (Figure 6). Notably, the model accurately adapts to the appropriate gravity magnitude over time in each case, despite no direct knowledge of the values of worldly parameters.

In Figure 3A, the adaptation from 1 to 0.5 *g* is shown, in response to an inputted sinusoidal, head-centered roll-tilt passive motion profile (amplitude, 30°; frequency, 0.25 Hz). This simple gravitational adaptation scenario demonstrates the shift in the posterior probability distribution over time, as well as the progression of the MMSE estimate that the Central Observer Model uses to produce orientation perception estimations. See Supplementary Figure 1 for how the sensory conflict signals respond under several of the parallel, alternative hypotheses, before and after the gravity transition, driving the model’s adaptation in the process. Figure 3B shows the perceived vs. the true motion profiles at four time epochs in the adaptation process. The model results suggest that misperceptions of orientation occur in the period after the gravitational shift, and before full adaptation to the new gravity level (time points T2 and T3, respectively). Future empirical studies could quantify spatial orientation perception using psychophysical tasks during gravity transitions, which would enable validation or falsification of these model predictions.

Figure 4 demonstrates predictions for three different gravitational transitions: 1*–*0.5 *g*, 1 *–*0.2 *g*, and 1–1.5 *g*, indicating the model’s ability to adapt to both hypo- and hyper- *g* scenarios. Each simulation was conducted with an inputted sinusoidal, head-centered roll-tilt motion profile (amplitude, 30°; frequency, 0.25 Hz). These simulations suggest that adaptation rates depend on the gravity level to which the body is transitioning, with larger hypo-*g* transitions leading to faster adaptation (1–0.2 *g* vs. 1–0.5 *g*) and hyper-g transitions requiring more time for full adaptation (1–1.5 *g* vs. 1–0.5 *g*). While these are just examples, see the “Discussion” section for an elaboration on the *g-*levels chosen for these simulations.

The inputted motion profile is also predicted to play a role in the model’s adaptation rate. Figure 5 shows the effects of various passive motion profiles, each with a gravitational transition from 1 to 0.5 *g*. More dynamic (i.e., higher amplitude or higher frequency) sinusoidal, head-centered roll-tilt profiles appear to adapt faster than less dynamic profiles, and any roll-tilt motion leads to faster adaptation relative to no motion at all (Figures 5A,B). However, the model predicts that varying amplitudes and magnitudes of lateral translation (Figure 5C) and upright yaw rotation (Figure 5D)—in which the CNS is receiving *either* otolith or semicircular canal cues that do not change orientation relative to gravity—do not have a substantial effect on adaptation rates.

Additionally, the distribution of prior probabilities influences the model’s adaptation rates. In Figures 6A,B, adaptation to 0.5 *g* is shown in two scenarios: a direct adaptation between gravity levels and learned adaptation following a series of gravitational transitions. The learned adaptation scenario results in a wider gravitational probability distribution, leading to a faster adaptation initialization, but a slower convergence to the true gravity level relative to the direct adaptation profile (Figure 6B).

**FIGURE 7:**
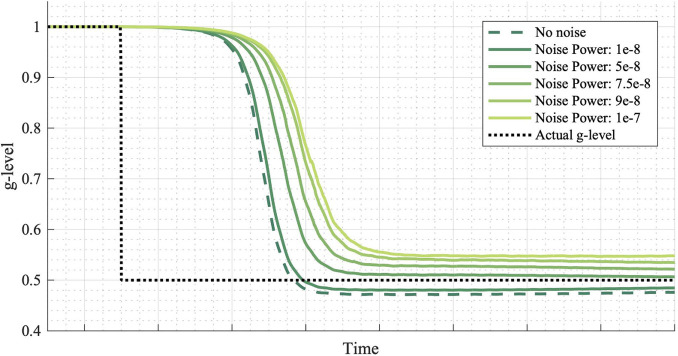
Effects of noise power on adaptation rates. Noise is added to the incoming measurements at various power levels. Higher noise powers result in slower adaptation initialization, though not necessarily slower convergence to the exact g-level. Inputted passive motion profiles were sinusoidal, head-centered roll-tilt (amplitude, 15°; frequency, 0.125 Hz).

Finally, Figure 7 shows the effect of noise power on simulation results. Noise power is defined as the height of the power spectral density of the white noise added to the system and may be thought of as an individual with more or less noisy vestibular sensors. More noise leads to a slower adaptation initialization. However, final convergence to the true gravity level does not follow the same trend. Note that a noise power of 1E-8 was used for the simulations in Figures 3–6. See the “Discussion” section for elaboration on the implications of differing noise levels.

Several assumptions were made while completing these simulations. First, twenty discrete alternative hypotheses ranging from 0.1 to 2.0 *g* (in steps of 0.1 *g*) were considered at each time step and remain unchanged through the simulations. Changes to the number of hypotheses or the hypotheses themselves are possible, but not shown (see section “Discussion”). Within these hypotheses, we implemented a probability “floor” to avoid any alternative effectively reaching zero probability, from which no evidence could build future support using Bayes rule (see Supplementary Table 1). Second, each of the gravity transitions were assumed instantaneous. Finally, for simplicity the model assumes no active motion or sensory input other than vestibular cues.

## Discussion

### Summary of Results and Contributions

To summarize, here we have proposed and implemented a computational model for how the CNS adapts to transitions in the magnitude of gravity, in terms of passive self-motion and orientation perceptual processing. Specifically, the model posits that the CNS uses alternative, parallel hypotheses for internal estimates of parameters of interest (here the internal estimate of the magnitude of gravity). Building upon the “observer” model framework, the “sensory conflict” signals (i.e., difference between sensory measurements and those expected by the CNS) for each alternative hypothesis were computed. Conceptually, the alternative hypothesis for which the internal magnitude of gravity is closest to the actual gravity magnitude will tend to produce the least sensory conflict, limited by sensory noise. Computationally, the multiple sensory conflict signals (**e_a_**, **e_f_**, **e**_*ω*_), each of which are three dimensional, were combined and normalized using the NIS statistic to yield a unidimensional metric. The likelihood of the error corresponding to each NIS value for each alternative hypothesis was calculated and used in Bayes rule to sequentially update the posterior probability of each alternative hypothesis being true. The final Central Observer Model representing the CNS’s accepted internal state parameters was estimated with an MMSE estimate across alternative hypotheses. Through a series of computations such as these proposed, it is possible for the CNS to adapt to changes in the magnitude of gravity. While alternatives (e.g., using a Kalman filter model, avoiding Bayes rule) are plausible, to our knowledge this is the first implementation capable of capturing adaptation of the spatial orientation system to altered gravity.

In order to demonstrate the model’s functionality and produce quantitative predictions, we implemented and then simulated the model with various scenarios. This implementation makes some important quantitative predictions, which are summarized here. Future experimental work will need to be performed in order to validate (or invalidate) the model predictions. In each case, the internal magnitude of gravity predicted by the model presumably cannot be directly measured empirically. Instead, perceptions of motion (e.g., tilt perception) can be assessed using psychophysical tasks (Clark et al., 2015b; Clark and Young, 2017; Galvan-Garza et al., 2018), during and following gravity transitions, in order to validate model predictions. Transitions to hyper-gravity (Schone, 1964; Clark et al., 2015a,b) can be performed using human-rated centrifuges. Alternative gravitational analogs such as parabolic flights (Clark and Young, 2017; Meskers et al., 2021), a supine centrifuge “hypo-gravity” paradigm (Galvan-Garza et al., 2018), or the Wheelchair Head Immobilization Paradigm (Dixon and Clark, 2020) (single-plane micro-gravity analog) could also be utilized to explore various aspects of the model’s predictions. Conceptually, the model predicts overestimation of tilt when the internal magnitude of gravity is too low (e.g., transitioning to hyper-gravity) and underestimation when it is too high. Tilt perception can be assessed using common psychophysical tasks, such as subjective visual vertical (SVV).

Most importantly, simulations show the model is capable of adapting to various altered gravity environments (e.g., 0.5, 0.2, or 1.5 *g* in Figure 4). This is accomplished despite the model (and the CNS) having no direct knowledge or input of the actual magnitude of gravity. Instead, the sensory measurements (from the otoliths and semicircular canals) are processed with the alternative hypotheses of the internal magnitude of gravity using parallel observer models to produce sensory conflict signals. Those which produce the least sensory conflict have the highest likelihood probability and using Bayes rule sequentially increases the posterior probabilities of the alternative hypothesis that are closest to the actual magnitude of gravity.

#### Time Course of Adaptation

Notably, this adaptation process does not occur instantaneously. In fact, immediately following a gravity transition the internal best estimate of the magnitude of gravity is unchanged, which results in misperceptions of orientation (as observed empirically; Clement and Wood, 2014; Clark et al., 2015b; Clark and Young, 2017). Only after evidence (in the form of sensory measurements producing sensory conflicts) has accumulated does the internal magnitude of gravity begin to change, slowly transitioning toward the actual gravity level and eventually converging. As seen in Figure 3, this transition follows a sigmoidal-shaped pattern over time, due to the probability distribution at the instant of the gravity transition being tightly centered about the previous magnitude of gravity. Initially there is only a very slow change in the best estimate of the internal magnitude of gravity, but over time (T3 in Figure 3) a bimodal distribution is produced with peaks near the previous actual magnitude (due to prior probabilities) and the new one (due to accumulating evidence updating the posterior probability). Once this second peak in the distribution has developed, then rather quickly the probability of the new magnitude of gravity becomes dominant and the best estimate of the internal magnitude of gravity converges to the new value.

How quickly this transition occurs, the model predicts, depends upon several factors. First, it predicts that adaptation occurs more quickly for larger gravity transitions (e.g., 1–0.2 *g* in Figure 4B is faster than 1–0.5 *g* in Figure 4A). At first this seems counterintuitive, since conceptually a larger gravity transition might require “more” adaptation. In contrast, in the model a larger gravity transition produces sensory measurements that are less consistent with the original magnitude of gravity (e.g., measurements after transitioning to 0.2 *g* are highly inconsistent with the alternative hypothesis of the magnitude of gravity still being 1 *g*, while transitioning to 0.5 *g* produces less inconsistent sensory measurements). This larger sensory conflict associated with the larger gravity transition drives adaptation more quickly. This may have implications for clinical conditions with more gradual changes (e.g., acoustic neuromas) vs. more rapid (e.g., labyrinthitis). Further, the model predicts that an adaptation to 1.5 *g* occurs more slowly than 1–0.5 *g* (Figures 4A,C), despite the equal magnitude in the change in gravity. We should note that this asymmetry in adapting to hyper-gravity vs. hypo-gravity is dependent upon the roll tilt profile experienced during the adaptation period. If the simulations are performed with no physical motion (simulations not shown), then the adaptation process is essentially symmetric. To our knowledge, these predictions have not been experimentally tested in a manner that could validate or invalidate them. We suggest a key benefit of implementing a computational model is to discover specific (potentially counterintuitive) quantitative predictions not yet hypothesized, to inform future experiments.

Next, we found the model predicts the rate of adaptation is dependent upon the motions experienced during and following the gravity transition. Specifically, larger and higher frequency tilt motions drive faster adaptation (Figures 5A,B). This prediction seems intuitive, and at least anecdotally has been observed by astronauts transitioning back to Earth gravity, who report that making head tilts helps adaptation more quickly than holding the head stable (Reschke and Clement, 2018). Within the model, this can be thought of as greater amplitude and higher frequency tilts increasing the signal (for constant sensory noise), making the gravity transition produce more obvious sensory evidence supporting a change in the internal magnitude of gravity. It should be mentioned that when making larger, higher frequency (i.e., faster) head tilts, the model predicts (as has been observed; Reschke and Clement, 2018) greater misperception of spatial orientation. Thus, large, rapid head tilts may expedite adaptation, but the model would suggest this should be done with caution to avoid misperceptions and sensorimotor impairment at critical mission phases, such as during planetary landings where manual control may be required. Interestingly, when the motion during a gravity transition is exclusively either lateral translation (Figure 5C) or upright yaw rotation (Figure 5D) (i.e., no tilt relative to gravity), the model predicts that adaptation will eventually occur, but that there is a negligible effect of the magnitude or frequency of these types of motions, compared to experiencing no motion. In the model, this is due to the fact that these non-tilt motions—where the otolith and semicircular canal cues are uninformative regarding the magnitude of gravity—produce no additional informative sensory conflict to update posterior probability and drive adaptation. Again, we are unaware of experimental evidence testing this, but the very specific quantitative predictions encourage such empirical assessment.

In Figure 6, we compared adaptation rates for the same transition (using the same motion stimuli), but in one case the transition was preceded by a series of other gravity transitions (learned adaptation). The effect of these earlier gravity transitions was a larger spread in the prior probability distribution across a wider range of alternative hypotheses for the internal magnitude of gravity. Essentially, the repeated gravity transitions produced less internal certainty in the gravity magnitude. This had an interesting effect on the final gravity transition, in which adaptation was initiated faster, but then took longer to converge upon the actual magnitude of gravity post-transition. This is an interesting prediction in the context of the concept of “learning to learn,” in which recent adaptations have been observed to enable faster subsequent adaptations, even to novel transitions (Roller et al., 2001, 2009; Seidler, 2004; Batson et al., 2011). While there is empirical evidence of “learning to learn” benefits specific to adapting to novel gravity transitions (Clark et al., 2015a), future studies should aim to quantify the early vs. late adaptation rates (as opposed to just overall) given these interesting model predictions. In particular, the model and experiments could explore whether previous adaptation to hyper-gravity, for example, benefit subsequent adaptation to hypo-gravity.

Finally, we considered a series of simulations of a simple gravity transition, but in which the sensory noise varied (Figure 7). Vestibular perceptual thresholds are thought to primarily be a measure of vestibular sensory noise, such that an individual with higher sensory noise will have higher thresholds (Nouri and Karmali, 2018; Diaz-Artiles and Karmali, 2021). Furthermore, vestibular perceptual thresholds vary dramatically as a function of aging (Bermudez Rey et al., 2016; Karmali et al., 2017), as well as between individuals of the same age (Grabherr et al., 2008; Valko et al., 2012; Lim et al., 2017; Suri and Clark, 2020). Similarly, there are substantial inter-individual differences in capacity to adaptat to altered gravity (Seidler et al., 2015). It has previously been suggested that vestibular sensory noise may be a limiting factor for sensorimotor adaptation (Clark and Merfeld, 2017), and this model prediction supports the idea that individuals with greater vestibular sensory noise may be slower to adapt to gravity transitions. Excitingly, there is some evidence that through extensive training, an individual may improve their innate vestibular perceptual thresholds (Klaus et al., 2020), though whether that would in turn improve their capacity to adapt to altered gravity remains untested.

As noted, many of these quantitative model predictions have not previously been tested empirically, so it is difficult to assess the validity of the model. However, the major predictions of this model implementation are consistent with existing data: the CNS eventually adapts to any gravity transition without any explicit knowledge of the actual magnitude of gravity. Further, this adaptation can be expedited through large amplitude, higher frequency (i.e., faster) head tilts. The remaining model predictions remain to be validated, but critically our simulations here provide novel quantitative hypotheses to motivate future experimental investigation. To date, this is the first model to be implemented for the computations that may be required by the CNS to adapt the orientation perception system to gravity transitions.

### Limitations and Future Work

In our simulations, we have focused on gravity transitions between 1 *g*, hypo-gravity (e.g., 0.5 *g*) and hyper-gravity (e.g., 1.5 *g*). Notably, we have not included any simulations to or from microgravity (i.e., 0 *g*), such as astronauts experience during missions to the ISS or in transit to the moon or in the future to Mars. We suggest that the adaptation to 0 *g* is somewhat unique and may involve the CNS making more than just an adjustment to the internal magnitude of gravity (Clark, 2019). For example, it has been hypothesized that long duration microgravity exposure causes the CNS to reinterpret all otolith stimulation as being due to translation (as opposed to tilts) (Young et al., 1984; Parker et al., 1985), or that an internal model for how rotational cues influence tilt perception degrades (Merfeld, 2003). These conceptual hypotheses may be captured in our computational model by expanding the set of alternative hypotheses to include not just various values for the internal magnitude of gravity, but also allow for different values for other parameters within each parallel observer model. In this case, the same computations of computing sensory conflict signals, collapsing to a unidimensional measure using NIS, calculating the error likelihood, and sequentially applying Bayes rule could all be applied, as we have already implemented.

Closely related to this limitation is that in our implementation we only assumed a fixed set of discrete alternative hypotheses of the internal magnitude of gravity. Even when simplifying that the parallel, alternative hypotheses only consider the one parameter of the internal magnitude of gravity, this is likely another (over)simplification to assume the CNS uses a fixed set of discrete alternatives (e.g., 0.1, 0.2, 0.3 *g*, etc.). Computationally, it was simpler to assume these alternatives were fixed, but biologically this would be a poor implementation. First, by discretizing in increments of 0.1 *g* between 0.1 and 2 *g*, means the model is incapable of adapting to gravity levels greater than 2 *g*. We could, of course, include more alternatives up to higher gravity levels (e.g., 3 *g*), but this requires the model (and the CNS) to know what gravity levels are possible to encounter in the first place. Second, it is computationally inefficient to have to process alternative hypotheses that do not ever occur. For example, in a gravity transition from 1 to 0.5 *g*, all of the alternatives between 1.3 and 2 *g* are effectively unused, but still require extensive computations.

Fortunately, a more intelligent, adaptive approach for dynamically selecting alternative hypotheses for the internal magnitude of gravity is possible. In a Rao-Blackwellized particle filter (Ristic et al., 2003), alternative hypotheses are resampled based upon the probability of previous alternatives. In our application, conceptually this amounts to dynamically creating alternative hypotheses for the internal magnitude of gravity, based upon having a higher prior probability distribution and then assessing their error likelihood. This process is done iteratively over time, allowing new alternatives to be spawned when they may be likely, and others to be removed when they become highly unlikely. This has computational advantages by not computing sensory conflict for alternatives that are entirely unlikely, but also prioritizing finer discretization near values that are more likely to be the internal magnitude of gravity at a given point in time. Future work should compare predictions and computational burden of our current fixed discretization and a Rao-Blackwellized particle filter approach to resample alternative hypotheses for the internal magnitude of gravity. This can begin to explore how the CNS *learns* new alternative hypotheses, as opposed to simply *adapting* between preexisting alternatives. The creation of new alternative hypotheses within this framework is scientifically interesting for scenarios in which the person must adapt to a novel environment entirely different from those previously experienced, such as first-time astronauts entering microgravity. In addition, this formulation circumvents the need to have a “floor” probability for each alternative hypothesis, since these are dynamically added/removed.

Next, to yield central perceptions of orientation during adaptation, we computed the MMSE estimate of the fixed, discrete alternatives to determine the internal magnitude of gravity within the final Central Observer Model. This is represented by the dotted red line in Figure 3A. However, there are other estimators of central tendency (e.g., maximum likelihood estimator) that could be used. Particularly, since the probability distributions for the internal magnitude of gravity (particularly during gravity transitions) are often bimodal, other approaches are reasonable to consider. Ideally, empirical data could help guide whether the adaptation process is smooth (as predicted with the MMSE estimator) or more of an abrupt switch from one alternative to another (as a maximum *a posteriori* estimator would produce, since it would just use whichever single discrete hypothesis has the highest posterior probability at each point of time).

Our model aims to implement the cognitive-based Computations for Orientation and Motion Perception in Altered Sensorimotor States (COMPASS) that may be required of the CNS to perform to adapt to gravity transitions. However, where those computations may occur in the brain or how neural networks and neurotransmitters may perform those computations are both questions beyond the scope of this work. Briefly, we note that the cerebellum is known to be heavily involved with internal models (Wolpert et al., 1998; Ito, 2000), including those related to orientation perception (Brooks and Cullen, 2013; Shaikh et al., 2013; Laurens and Angelaki, 2016). Further, the computations required within our model (additions and multiplications) are likely feasible using multilayer, neural networks.

While here we focused on adaptation to altered environments, this same computational model framework can be used to study other adaptations to orientation perception processing. For example, aging is known to alter sensory transduction (Karmali et al., 2018) and the brain appears to update its processing consistent with static Bayesian inference, but the dynamic adaptation (i.e., over time with aging) to these changes has not been computationally modeled. Similarly, how the CNS reinterprets acute damage to peripheral vestibular sensors could be modeled computationally using our framework. Broadly, the proposed computational model can be applied to various scenarios of sensorimotor adaptation or learning by reconsidering the parameter(s) which differ between alternative hypotheses resulting in various sets of sensory conflict signals, and then performing error likelihood and Bayes rule computations to update posterior probabilities across the alternative, parallel hypotheses to produce a central model used for perception and action.

## Data Availability Statement

The raw data supporting the conclusions of this article will be made available by the authors, without undue reservation.

## Author Contributions

TC, NA, JD, and VK contributed to the conception of the project, the functionality of the computational model, and reviewing of simulation results. JD and VK implemented the model. TC, JD, and VK wrote sections of the manuscript. All authors contributed to manuscript revision, read, and approved the submitted version.

## Conflict of Interest

The authors declare that the research was conducted in the absence of any commercial or financial relationships that could be construed as a potential conflict of interest.

## Publisher’s Note

All claims expressed in this article are solely those of the authors and do not necessarily represent those of their affiliated organizations, or those of the publisher, the editors and the reviewers. Any product that may be evaluated in this article, or claim that may be made by its manufacturer, is not guaranteed or endorsed by the publisher.
