# Supplementary material for: COMPASS: Computations for Orientation and Motion Perception in Altered Sensorimotor States
Source: Front Neural Circuits. 2021 Oct 15;15:757817. doi: 10.3389/fncir.2021.757817 (PMC8553968; doi:10.3389/fncir.2021.757817)
Supplement: Supplementary file 1 [file Data_Sheet_1.PDF]

Supplementary Material

Supplementary Table 1. Model Parameters. K_a, K_f, $K_{f\omega}$, and K_ω represent feedback gains in each observer model. K_{au} and $K_{au\perp}$ account for differential weighting in versus perpendicular to the utricle plane, appropriate to produce the g-excess illusion in hyper-gravity scenarios. σ_a, σ_f, and σ_ω represent the variance of the measurement noise. K_s is the gain for the covariance matrix, **S**, used in the NIS calculation. A computational floor is set to ensure that no gravitational hypotheses reach a probability of zero, thus becoming obsolete in future Bayesian probability update steps. Prior probability initialization values are arbitrary, as the values equilibrate over the beginning of each simulation, but approximate a 1 g -acclimated person. The initial values used for all simulations starting at 1 g are listed in the last row.

Model Parameters	Value
$K_{au\perp}$*	-4 [unitless]
K_{au}**	-2 [unitless]
K_f*	4 [1/seconds]
$K_{f\omega}$*	8 [1/seconds]
K_ω*	8 [unitless]
σ_a***	0.0024 [g's]
σ_f	0.0001 [radians]
σ_ω	0.003 [radians/second]
K_s	200,000 [unitless]
Computational floor (i.e., lowest possible probability)	1E-8 [probability]
Initialization prior probabilities for gravity hypotheses (0.1g:0.1g:2g)	[0.001, 0.002, 0.003, 0.004, 0.005, 0.006, 0.007, 0.008, 0.015, 0.897, 0.015, 0.008, 0.007, 0.006, 0.005, 0.004, 0.003, 0.002, 0.001, 0.001]

*Clark et al. 2019b
**Clark et al. 2015b

***Karmali & Merfeld 2012

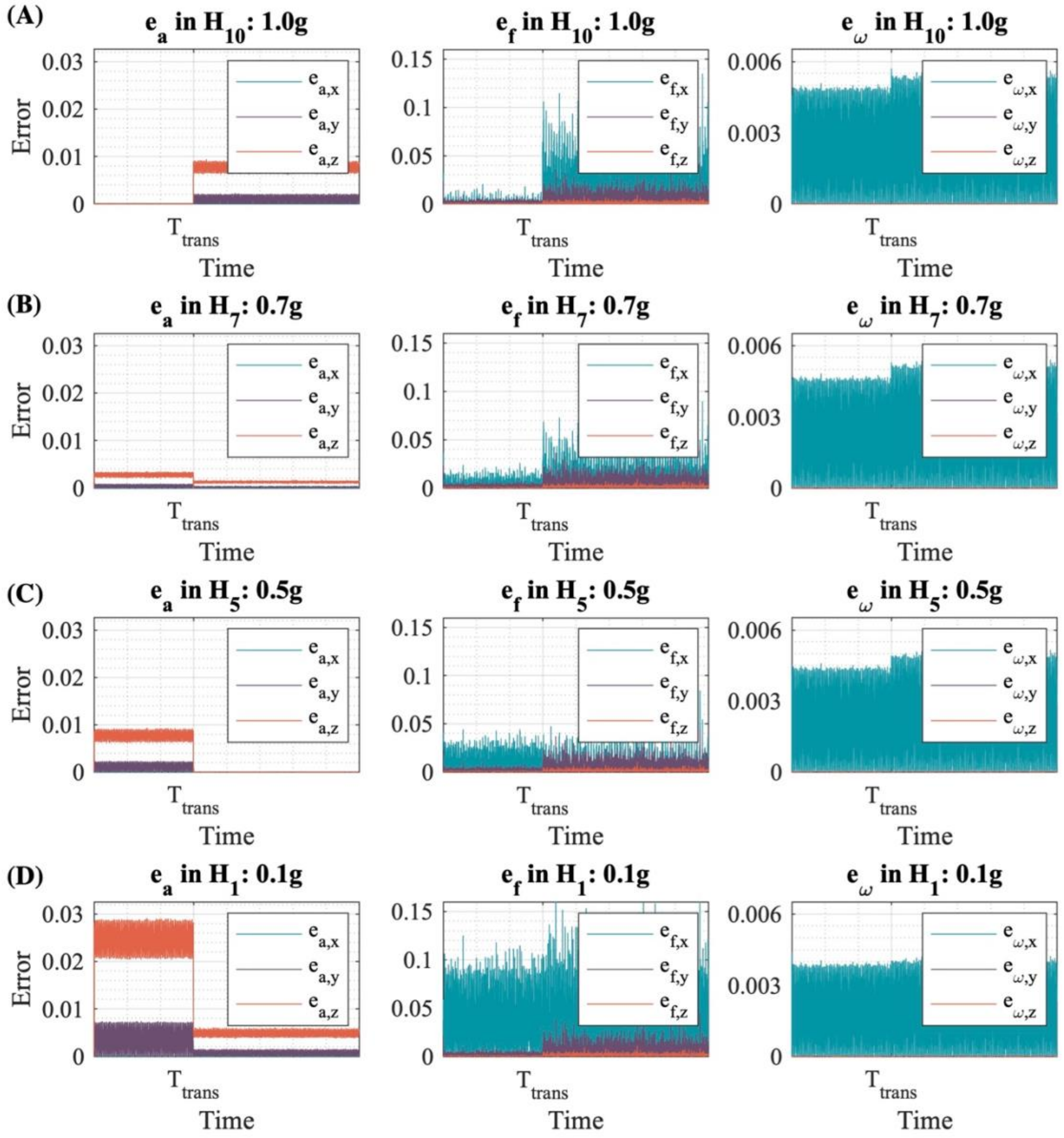

Supplementary Figure 1. Sensory conflict over time in several of the parallel observers (rows). The true gravity level transitions from 1.0 *g* to 0.5 *g* at T_{trans}, and incoming measurements follow passive, sinusoidal, head-centered roll-tilt (amplitude, 30°; frequency, 0.25Hz). Each three-dimensional signal has been processed through the NIS function (i.e., square and weighted by their diagonal element in the inverted **S** matrix) but remain as three unidimensional values here to show their relative contribution to the overall NIS statistic used to compute the likelihood for that hypothesis. **(A)** Contributions of $\boldsymbol{e_a}$, $\boldsymbol{e_f}$, and $\boldsymbol{e_\omega}$ to the total sensory conflict signal generated by the model hypothesizing $|\widehat{\boldsymbol{g}}| = 1.0\ g$. The error increases following the gravity transition, driving the model to

search for a better alternative. **(B)** Contributions of $\boldsymbol{e_a}$, $\boldsymbol{e_f}$, and $\boldsymbol{e_\omega}$ to the total sensory conflict signal generated by the model hypothesizing $|\hat{\boldsymbol{g}}|$ = 0.7 *g*. **(C)** Contributions of $\boldsymbol{e_a}$, $\boldsymbol{e_f}$, and $\boldsymbol{e_\omega}$ to the total sensory conflict signal generated by the model hypothesizing $|\hat{\boldsymbol{g}}|$ = 0.5 *g*. Following the gravity transition, the total error in this observer model decreases. **(D)** Contributions of $\boldsymbol{e_a}$, $\boldsymbol{e_f}$, and $\boldsymbol{e_\omega}$ to the total sensory conflict signal generated by the model hypothesizing $|\hat{\boldsymbol{g}}|$ = 0.1 *g*.
